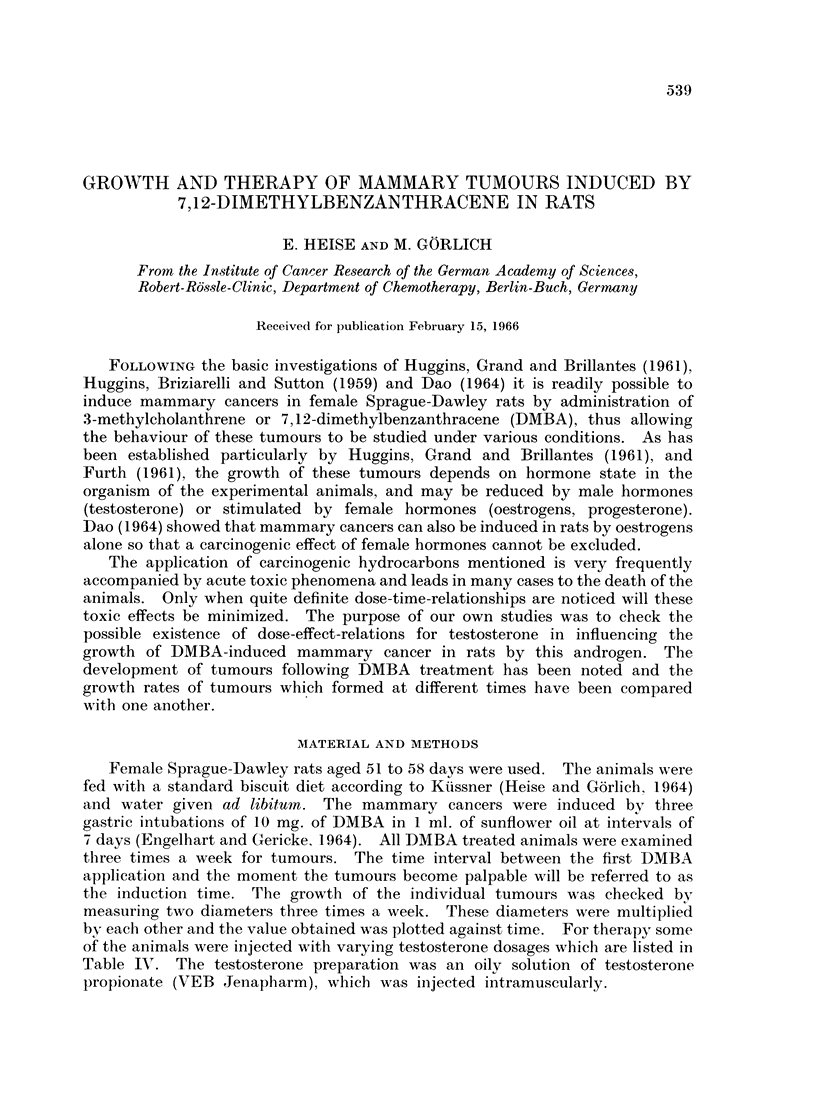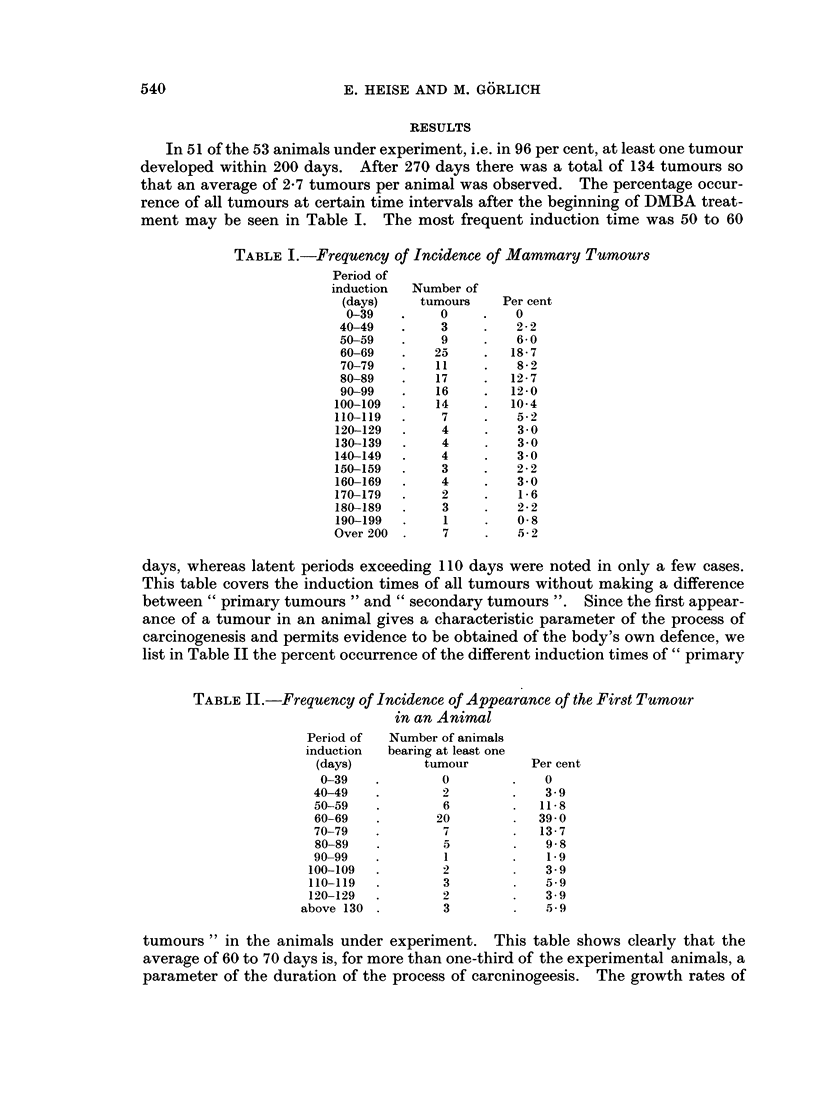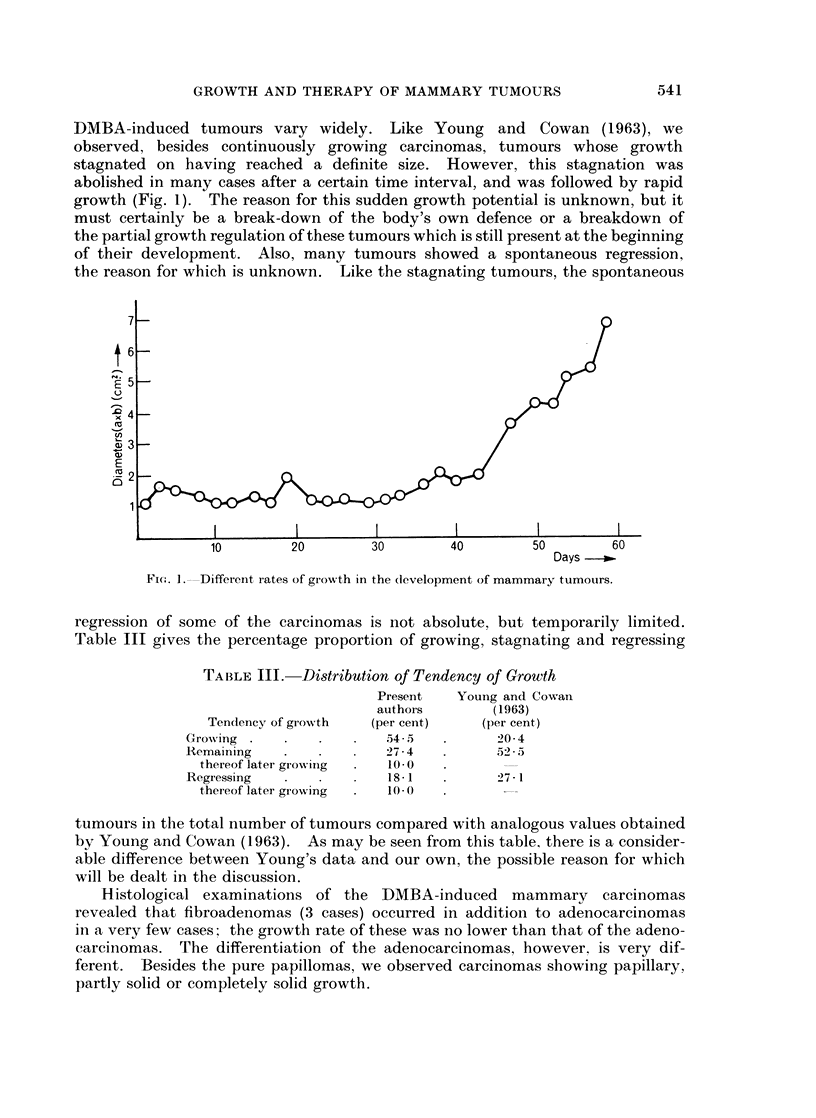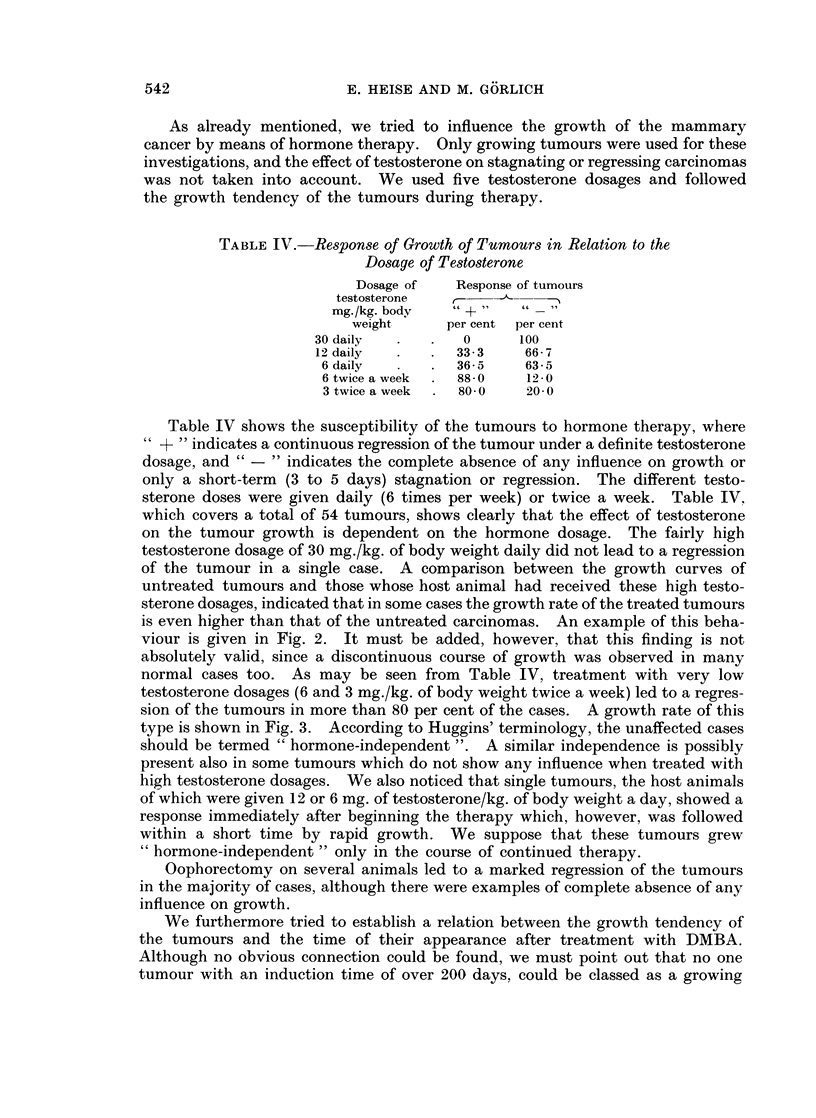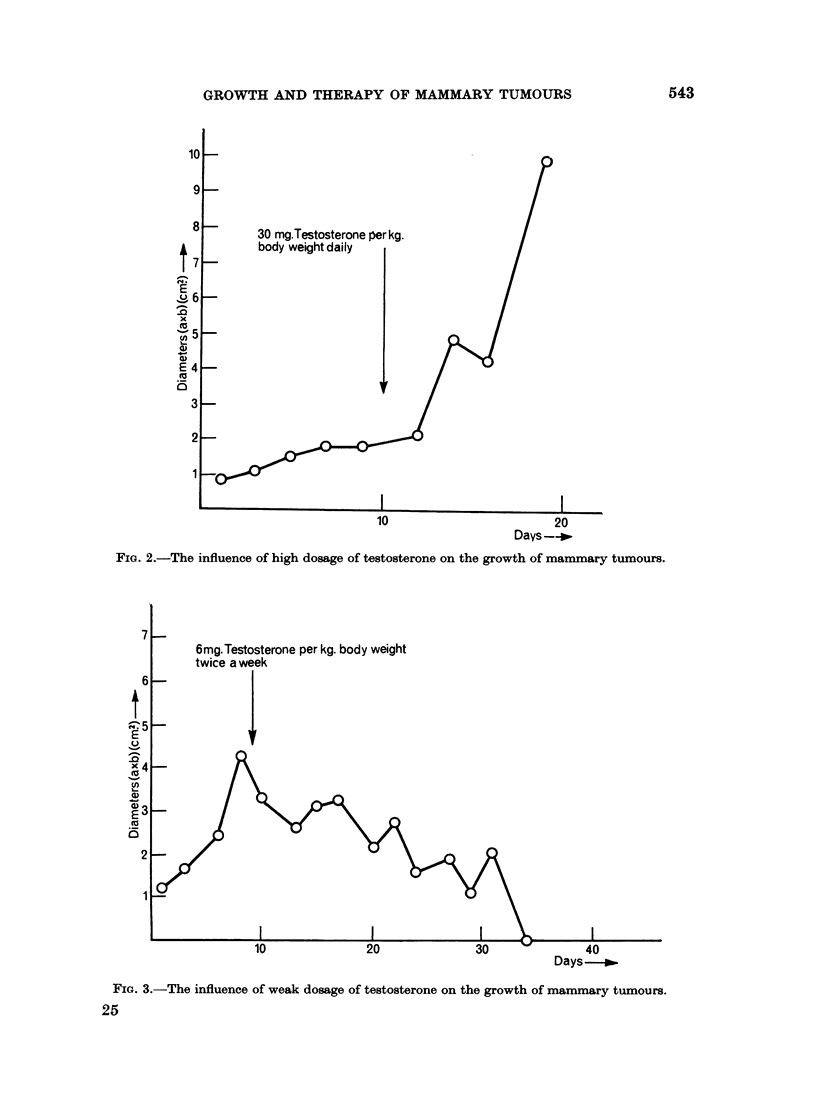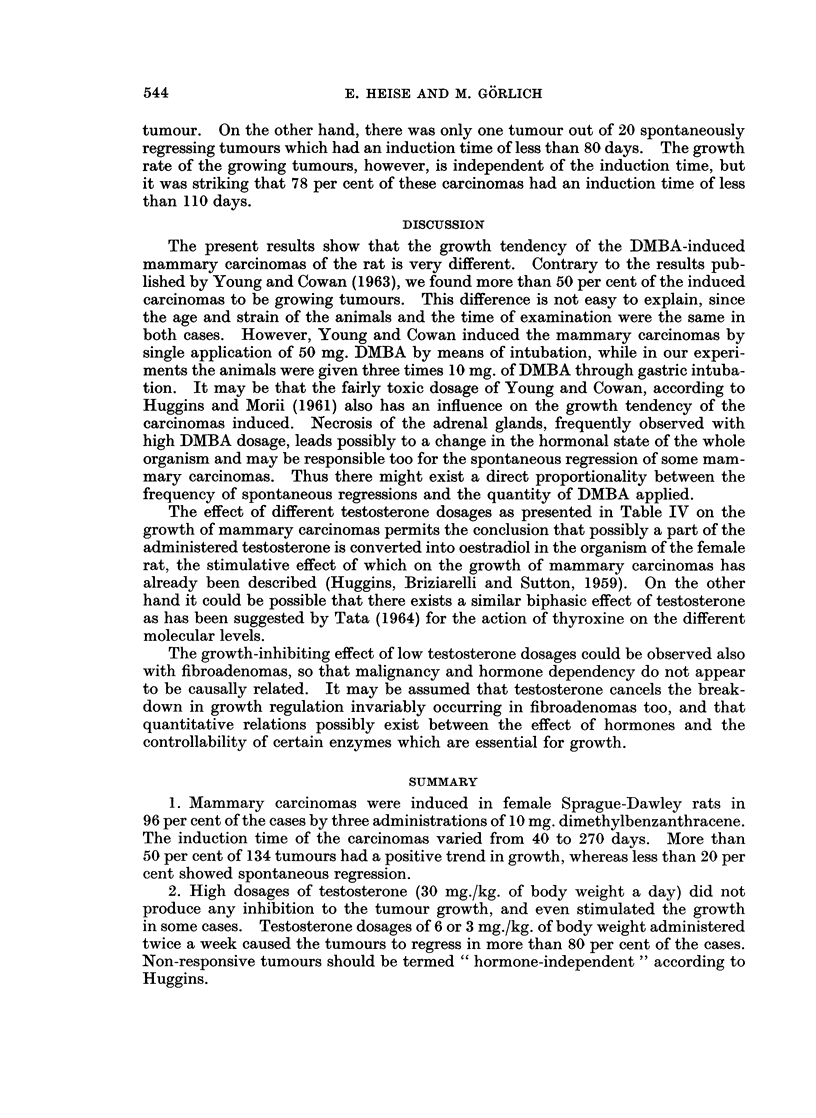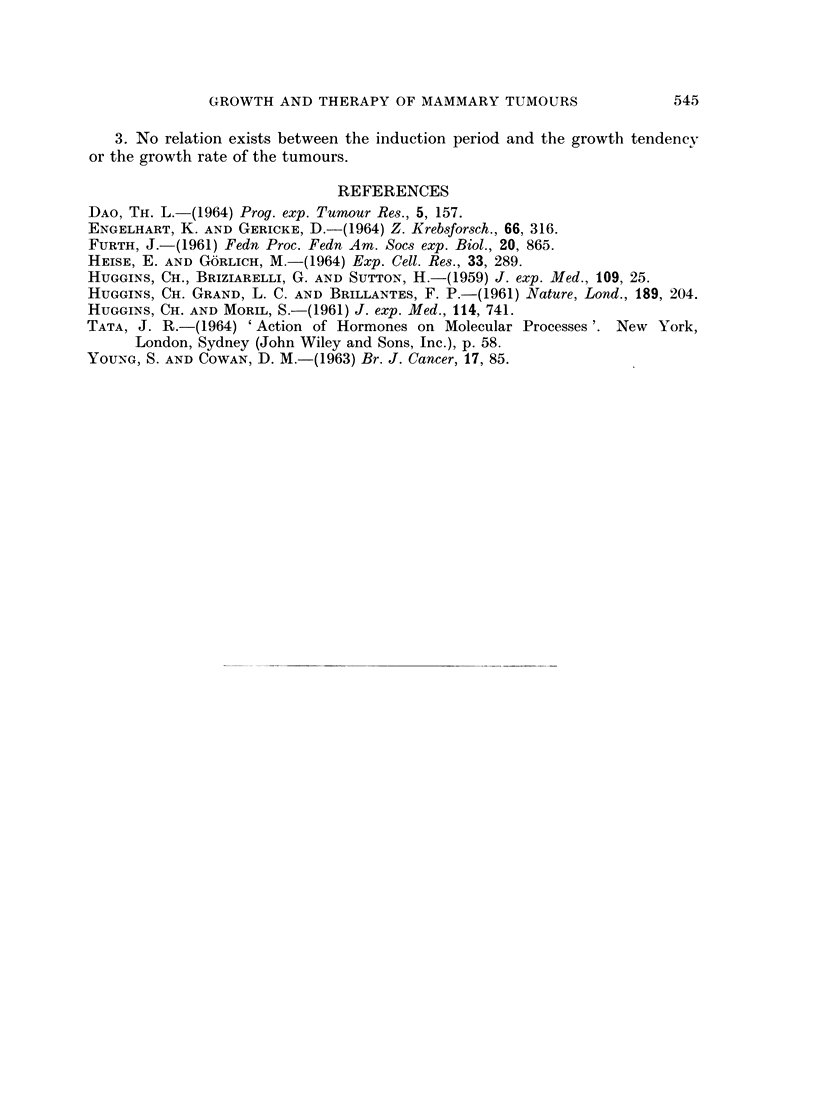# Growth and therapy of mammary tumours induced by 7,12-dimethylbenzanthracene in rats.

**DOI:** 10.1038/bjc.1966.66

**Published:** 1966-09

**Authors:** E. Heise, M. Görlich


### GROWTH AND THERAPY OF MAMMARY TUMOURS INDUCED BY 7,12-DIMETHYLBENZANTHRACENE IN RATS

E. HEISE AND M. GÖRLICH

*From the Institute of Cancer Research of the German Academy of Sciences, Robert-Rössle-Clinic, Department of Chemotherapy, Berlin-Buch, Germany*

Received for publication February 15, 1966

FOLLOWING the basic investigations of Huggins, Grand and Brillantes (1961), Huggins, Briziarelli and Sutton (1959) and Dao (1964) it is readily possible to induce mammary cancers in female Sprague-Dawley rats by administration of 3-methylcholanthrene or 7,12-dimethylbenzanthracene (DMBA), thus allowing the behaviour of these tumours to be studied under various conditions. As has been established particularly by Huggins, Grand and Brillantes (1961), and Furth (1961), the growth of these tumours depends on hormone state in the organism of the experimental animals, and may be reduced by male hormones (testosterone) or stimulated by female hormones (oestrogens, progesterone). Dao (1964) showed that mammary cancers can also be induced in rats by oestrogens alone so that a carcinogenic effect of female hormones cannot be excluded.

The application of carcinogenic hydrocarbons mentioned is very frequently accompanied by acute toxic phenomena and leads in many cases to the death of the animals. Only when quite definite dose-time-relationships are noticed will these toxic effects be minimized. The purpose of our own studies was to check the possible existence of dose-effect-relations for testosterone in influencing the growth of DMBA-induced mammary cancer in rats by this androgen. The development of tumours following DMBA treatment has been noted and the growth rates of tumours which formed at different times have been compared with one another.

### MATERIAL AND METHODS

Female Sprague-Dawley rats aged 51 to 58 days were used. The animals were fed with a standard biscuit diet according to Küssner (Heise and Görlich, 1964) and water given *ad libitum*. The mammary cancers were induced by three gastric intubations of 10 mg. of DMBA in 1 ml. of sunflower oil at intervals of 7 days (Engelhart and Gericke, 1964). All DMBA treated animals were examined three times a week for tumours. The time interval between the first DMBA application and the moment the tumours become palpable will be referred to as the induction time. The growth of the individual tumours was checked by measuring two diameters three times a week. These diameters were multiplied by each other and the value obtained was plotted against time. For therapy some of the animals were injected with varying testosterone dosages which are listed in Table IV. The testosterone preparation was an oily solution of testosterone propionate (VEB Jenapharm), which was injected intramuscularly.

### RESULTS

In 51 of the 53 animals under experiment, i.e. in 96 per cent, at least one tumour developed within 200 days. After 270 days there was a total of 134 tumours so that an average of 2·7 tumours per animal was observed. The percentage occurrence of all tumours at certain time intervals after the beginning of DMBA treatment may be seen in Table I. The most frequent induction time was 50 to 60 days, whereas latent periods exceeding 110 days were noted in only a few cases. This table covers the induction times of all tumours without making a difference between " primary tumours " and " secondary tumours ". Since the first appearance of a tumour in an animal gives a characteristic parameter of the process of carcinogenesis and permits evidence to be obtained of the body's own defence, we list in Table II the percent occurrence of the different induction times of " primary tumours " in the animals under experiment. This table shows clearly that the average of 60 to 70 days is, for more than one-third of the experimental animals, a parameter of the duration of the process of carcninogeesis. The growth rates of

TABLE I.—*Frequency of Incidence of Mammary Tumours*

| Period of induction (days) | Number of tumours | Per cent |
|---|---|---|
| 0–39 | 0 | 0 |
| 40–49 | 3 | 2·2 |
| 50–59 | 9 | 6·0 |
| 60–69 | 25 | 18·7 |
| 70–79 | 11 | 8·2 |
| 80–89 | 17 | 12·7 |
| 90–99 | 16 | 12·0 |
| 100–109 | 14 | 10·4 |
| 110–119 | 7 | 5·2 |
| 120–129 | 4 | 3·0 |
| 130–139 | 4 | 3·0 |
| 140–149 | 4 | 3·0 |
| 150–159 | 3 | 2·2 |
| 160–169 | 4 | 3·0 |
| 170–179 | 2 | 1·6 |
| 180–189 | 3 | 2·2 |
| 190–199 | 1 | 0·8 |
| Over 200 | 7 | 5·2 |

TABLE II.—*Frequency of Incidence of Appearance of the First Tumour in an Animal*

| Period of induction (days) | Number of animals bearing at least one tumour | Per cent |
|---|---|---|
| 0–39 | 0 | 0 |
| 40–49 | 2 | 3·9 |
| 50–59 | 6 | 11·8 |
| 60–69 | 20 | 39·0 |
| 70–79 | 7 | 13·7 |
| 80–89 | 5 | 9·8 |
| 90–99 | 1 | 1·9 |
| 100–109 | 2 | 3·9 |
| 110–119 | 3 | 5·9 |
| 120–129 | 2 | 3·9 |
| above 130 | 3 | 5·9 |

DMBA-induced tumours vary widely. Like Young and Cowan (1963), we observed, besides continuously growing carcinomas, tumours whose growth stagnated on having reached a definite size. However, this stagnation was abolished in many cases after a certain time interval, and was followed by rapid growth (Fig. 1). The reason for this sudden growth potential is unknown, but it must certainly be a break-down of the body's own defence or a breakdown of the partial growth regulation of these tumours which is still present at the beginning of their development. Also, many tumours showed a spontaneous regression, the reason for which is unknown. Like the stagnating tumours, the spontaneous

FIG. 1.—Different rates of growth in the development of mammary tumours.

regression of some of the carcinomas is not absolute, but temporarily limited. Table III gives the percentage proportion of growing, stagnating and regressing

TABLE III.—*Distribution of Tendency of Growth*

| Tendency of growth | Present authors (per cent) | Young and Cowan (1963) (per cent) |
|---|---|---|
| Growing . . . . | 54·5 | 20·4 |
| Remaining . . . | 27·4 | 52·5 |
| thereof later growing | 10·0 | — |
| Regressing . . . | 18·1 | 27·1 |
| thereof later growing | 10·0 | — |

tumours in the total number of tumours compared with analogous values obtained by Young and Cowan (1963). As may be seen from this table, there is a considerable difference between Young's data and our own, the possible reason for which will be dealt in the discussion.

Histological examinations of the DMBA-induced mammary carcinomas revealed that fibroadenomas (3 cases) occurred in addition to adenocarcinomas in a very few cases; the growth rate of these was no lower than that of the adenocarcinomas. The differentiation of the adenocarcinomas, however, is very different. Besides the pure papillomas, we observed carcinomas showing papillary, partly solid or completely solid growth.

As already mentioned, we tried to influence the growth of the mammary cancer by means of hormone therapy. Only growing tumours were used for these investigations, and the effect of testosterone on stagnating or regressing carcinomas was not taken into account. We used five testosterone dosages and followed the growth tendency of the tumours during therapy.

TABLE IV.—*Response of Growth of Tumours in Relation to the Dosage of Testosterone*

| Dosage of testosterone mg./kg. body weight | Response of tumours " + " per cent | " − " per cent |
| --- | --- | --- |
| 30 daily | 0 | 100 |
| 12 daily | 33·3 | 66·7 |
| 6 daily | 36·5 | 63·5 |
| 6 twice a week | 88·0 | 12·0 |
| 3 twice a week | 80·0 | 20·0 |

Table IV shows the susceptibility of the tumours to hormone therapy, where " + " indicates a continuous regression of the tumour under a definite testosterone dosage, and " — " indicates the complete absence of any influence on growth or only a short-term (3 to 5 days) stagnation or regression. The different testosterone doses were given daily (6 times per week) or twice a week. Table IV, which covers a total of 54 tumours, shows clearly that the effect of testosterone on the tumour growth is dependent on the hormone dosage. The fairly high testosterone dosage of 30 mg./kg. of body weight daily did not lead to a regression of the tumour in a single case. A comparison between the growth curves of untreated tumours and those whose host animal had received these high testosterone dosages, indicated that in some cases the growth rate of the treated tumours is even higher than that of the untreated carcinomas. An example of this behaviour is given in Fig. 2. It must be added, however, that this finding is not absolutely valid, since a discontinuous course of growth was observed in many normal cases too. As may be seen from Table IV, treatment with very low testosterone dosages (6 and 3 mg./kg. of body weight twice a week) led to a regression of the tumours in more than 80 per cent of the cases. A growth rate of this type is shown in Fig. 3. According to Huggins' terminology, the unaffected cases should be termed " hormone-independent ". A similar independence is possibly present also in some tumours which do not show any influence when treated with high testosterone dosages. We also noticed that single tumours, the host animals of which were given 12 or 6 mg. of testosterone/kg. of body weight a day, showed a response immediately after beginning the therapy which, however, was followed within a short time by rapid growth. We suppose that these tumours grew " hormone-independent " only in the course of continued therapy.

Oophorectomy on several animals led to a marked regression of the tumours in the majority of cases, although there were examples of complete absence of any influence on growth.

We furthermore tried to establish a relation between the growth tendency of the tumours and the time of their appearance after treatment with DMBA. Although no obvious connection could be found, we must point out that no one tumour with an induction time of over 200 days, could be classed as a growing

FIG. 2.—The influence of high dosage of testosterone on the growth of mammary tumours.

FIG. 3.—The influence of weak dosage of testosterone on the growth of mammary tumours.

tumour. On the other hand, there was only one tumour out of 20 spontaneously regressing tumours which had an induction time of less than 80 days. The growth rate of the growing tumours, however, is independent of the induction time, but it was striking that 78 per cent of these carcinomas had an induction time of less than 110 days.

### DISCUSSION

The present results show that the growth tendency of the DMBA-induced mammary carcinomas of the rat is very different. Contrary to the results published by Young and Cowan (1963), we found more than 50 per cent of the induced carcinomas to be growing tumours. This difference is not easy to explain, since the age and strain of the animals and the time of examination were the same in both cases. However, Young and Cowan induced the mammary carcinomas by single application of 50 mg. DMBA by means of intubation, while in our experiments the animals were given three times 10 mg. of DMBA through gastric intubation. It may be that the fairly toxic dosage of Young and Cowan, according to Huggins and Morii (1961) also has an influence on the growth tendency of the carcinomas induced. Necrosis of the adrenal glands, frequently observed with high DMBA dosage, leads possibly to a change in the hormonal state of the whole organism and may be responsible too for the spontaneous regression of some mammary carcinomas. Thus there might exist a direct proportionality between the frequency of spontaneous regressions and the quantity of DMBA applied.

The effect of different testosterone dosages as presented in Table IV on the growth of mammary carcinomas permits the conclusion that possibly a part of the administered testosterone is converted into oestradiol in the organism of the female rat, the stimulative effect of which on the growth of mammary carcinomas has already been described (Huggins, Briziarelli and Sutton, 1959). On the other hand it could be possible that there exists a similar biphasic effect of testosterone as has been suggested by Tata (1964) for the action of thyroxine on the different molecular levels.

The growth-inhibiting effect of low testosterone dosages could be observed also with fibroadenomas, so that malignancy and hormone dependency do not appear to be causally related. It may be assumed that testosterone cancels the breakdown in growth regulation invariably occurring in fibroadenomas too, and that quantitative relations possibly exist between the effect of hormones and the controllability of certain enzymes which are essential for growth.

### SUMMARY

1. Mammary carcinomas were induced in female Sprague-Dawley rats in 96 per cent of the cases by three administrations of 10 mg. dimethylbenzanthracene. The induction time of the carcinomas varied from 40 to 270 days. More than 50 per cent of 134 tumours had a positive trend in growth, whereas less than 20 per cent showed spontaneous regression.

2. High dosages of testosterone (30 mg./kg. of body weight a day) did not produce any inhibition to the tumour growth, and even stimulated the growth in some cases. Testosterone dosages of 6 or 3 mg./kg. of body weight administered twice a week caused the tumours to regress in more than 80 per cent of the cases. Non-responsive tumours should be termed " hormone-independent " according to Huggins.

3. No relation exists between the induction period and the growth tendency or the growth rate of the tumours.

### REFERENCES

DAO, TH. L.—(1964) *Prog. exp. Tumour Res.*, **5**, 157.
ENGELHART, K. AND GERICKE, D.—(1964) *Z. Krebsforsch.*, **66**, 316.
FURTH, J.—(1961) *Fedn Proc. Fedn Am. Socs exp. Biol.*, **20**, 865.
HEISE, E. AND GÖRLICH, M.—(1964) *Exp. Cell. Res.*, **33**, 289.
HUGGINS, CH., BRIZIARELLI, G. AND SUTTON, H.—(1959) *J. exp. Med.*, **109**, 25.
HUGGINS, CH. GRAND, L. C. AND BRILLANTES, F. P.—(1961) *Nature, Lond.*, **189**, 204.
HUGGINS, CH. AND MORIL, S.—(1961) *J. exp. Med.*, **114**, 741.
TATA, J. R.—(1964) 'Action of Hormones on Molecular Processes'. New York, London, Sydney (John Wiley and Sons, Inc.), p. 58.
YOUNG, S. AND COWAN, D. M.—(1963) *Br. J. Cancer*, **17**, 85.